# Factors Associated With Colleague Solidarity Among Hospital Nurses: Shame‐Proneness, Guilt‐Proneness, and Mentalization

**DOI:** 10.1155/jonm/4832980

**Published:** 2026-11-14

**Authors:** Jiyeon Kim, Hyojung Choi, Sun Joo Jang

**Affiliations:** ^1^ College of Nursing Seoul National University 103 Daehak-Ro, Jongno-Gu Seoul South Korea snu.ac.kr; ^2^ College of Nursing and The Research Institute of Nursing Science Seoul National University 103 Daehak-Ro, Jongno-Gu Seoul South Korea snu.ac.kr

**Keywords:** colleague solidarity, guilt-proneness, mentalization, nursing management, self-conscious emotions, shame-proneness

## Abstract

**Background:**

Although colleague solidarity among nurses supports mutual assistance and knowledge sharing, the psychological characteristics associated with it remain insufficiently understood.

**Aim:**

To examine the associations of shame‐proneness, guilt‐proneness, and mentalization with colleague solidarity among nurses in South Korean tertiary general hospitals, adjusting for demographic and work‐related characteristics.

**Methods:**

A cross‐sectional online survey was conducted from October 27 to December 31, 2025, with 223 nurses recruited by convenience sampling, of whom 205 working in South Korean tertiary general hospitals were eligible and analyzed. Participants completed the Test of Self‐Conscious Affect‐3 Short Form, Mentalization Scale, and Colleague Solidarity Scale. Data were analyzed using descriptive statistics, zero‐order and partial correlations, and hierarchical multiple regression adjusting for demographic and work‐related covariates, with multiple imputation for two missing item responses.

**Results:**

In the final model (adjusted *R* ^2^ = 0.22), mentalization (*β* = 0.29, 95% confidence interval [CI]: [0.11, 0.46], *p* = 0.001) and guilt‐proneness (*β* = 0.16, 95% CI: [0.04, 0.29], *p* = 0.007) were positively associated with colleague solidarity, whereas shame‐proneness was negatively associated (*β* = −0.25, 95% CI: [−0.40, −0.09], *p* = 0.002). The associations of mentalization and guilt‐proneness with solidarity were consistent across sensitivity analyses, whereas the association of shame‐proneness was not significant after adjustment for externalization and detachment tendencies.

**Conclusions:**

Nurses with higher mentalizing capacity and greater guilt‐proneness reported stronger colleague solidarity. Mentalization and self‐conscious emotional tendencies therefore merit attention as individual‐level correlates of solidarity, alongside organizational conditions.

**Implications for Nursing Management:**

Nurse managers could support colleague solidarity by promoting reflective practice that strengthens perspective‐taking and by helping nurses distinguish guilt about a specific action from self‐directed shame, within psychologically safe relationships. Such mentalization‐informed and shame‐sensitive approaches should be developed and evaluated alongside organizational improvements.

## 1. Introduction

Nurses working in tertiary general hospitals coordinate care for patients with complex needs. Relationships among nurses are therefore relevant to overall nursing performance and patient safety [1, 2]. While workload and interpersonal difficulties can complicate this coordination, colleague solidarity offers a way to examine the mutual support underlying nurses’ working relationships [3, 4].

Colleague solidarity, which involves respect, mutual support, and willingness to share professional knowledge and skills [4, 5], has been associated with organizational climate, work motivation, and job performance [3, 6, 7]. Understanding the psychological characteristics associated with solidarity may complement research on working conditions and help clarify how nurses perceive and respond to their colleagues. The organizational relevance of solidarity does not exhaust its meaning for individual nurses. In a qualitative secondary analysis of interviews with 13 Norwegian nurses, Kristoffersen described valued mutual dependence and experiences of limited sympathy within the same professional community [8]. This finding suggests that collegial relationships can remain important even when emotional support is inconsistent. It also cautions against assuming that solidarity is simply the absence of interpersonal conflict. Examining how nurses appraise themselves and understand colleagues may add to organizational accounts of solidarity without replacing attention to the conditions in which those relationships develop.

Shame and guilt are self‐conscious emotions that involve evaluating oneself against personal or societal standards. State shame centers on a negative evaluation of the self and may motivate withdrawal or concealment, whereas state guilt centers on a specific behavior and may motivate acknowledgment and repair [9, 10]. Shame‐proneness and guilt‐proneness describe tendencies to experience these responses across situations. The Test of Self‐Conscious Affect‐3 Short Form (TOSCA‐3S) assesses these tendencies through hypothetical scenarios [11]. Its shame responses emphasize maladaptive self‐evaluation and its guilt responses emphasize reparative behavior; guilt responses should not be equated with persistent, dysfunctional guilt. These differences between shame and guilt responses provide a rationale for anticipating negative and positive associations, respectively, with solidarity [12].

Shame‐related withdrawal may be incompatible with seeking support from or engaging with colleagues, whereas acknowledgment of a specific mistake may support relationship repair. These are theoretical expectations, rather than established mechanisms in the nursing profession. Research among South Korean nurses links self‐conscious emotions with workplace bullying [13], and workplace research links anticipated guilt with intentions to help coworkers [14]. As shame and guilt can co‐occur, examining each while controlling for the other may clarify their distinct associations [12].

Workplace studies offer a more specific rationale for distinguishing these emotional tendencies. A study on knowledge hiding examined guilt and shame as different responses to withholding information: guilt was linked to compensatory citizenship behavior, whereas shame was linked to withdrawal [15]. A survey of employees witnessing incivility similarly associated shame with avoidance, while guilt was associated with support for the target as well as retaliation against the instigator [16]. These studies concern reactions to particular events, and their outcomes differ from colleague solidarity. They therefore support the conceptual distinction between shame and guilt, while leaving open whether dispositional TOSCA‐3S scores show comparable associations with colleague solidarity among nurses.

Mentalization is the capacity to understand behavior in terms of mental states, including one’s own and others’ feelings, intentions, and beliefs [17, 18]. Difficulty understanding these states may complicate interpersonal relationships, whereas greater mentalizing capacity may support perspective‐taking and reflective responses to interpersonal difficulty [17, 19]. For nurses, the distinction is relevant when the same interaction can be interpreted as criticism, a request for help, or a response to clinical pressure. Self‐related reflection may help nurses recognize their own reactions, while attention to another person’s perspective may support consideration of alternative explanations. These are proposed relational processes, not established effects of mentalization on solidarity. The present study therefore examines mentalization alongside shame‐ and guilt‐proneness rather than treating mentalization as a demonstrated mediator between emotions and relationships.

Existing solidarity research has considered aspects such as organizational climate, work motivation, and job performance [3, 6, 7], while the psychological processes relevant to nurses’ everyday relationships remain less clearly delineated. We therefore examine the associations of shame‐proneness, guilt‐proneness, and mentalization with colleague solidarity among nurses working in South Korean tertiary general hospitals.

## 2. Materials and Methods

### 2.1. Study Design

This study uses a cross‐sectional design and is reported in accordance with the Strengthening the Reporting of Observational Studies in Epidemiology guidelines for cross‐sectional studies.

### 2.2. Participants and Data Collection

Inclusion criteria were being a nurse working in a tertiary general hospital in South Korea and having worked for more than 3 months at the time of the survey (i.e., having completed the probationary period). Exclusion criteria were being a nurse manager, working in a department staffed by only one nurse, or having a primarily administrative role. Sample size was estimated a priori using G∗Power 3.1.9.7 with *α* = 0.05, power = 0.95, *f* ^2^ = 0.15, and 16 predictor parameters, including dummy‐coded categorical variables, yielding a minimum of 204 participants. The 16 parameters comprised two indicators for age, one each for sex and religion, two for education, three for clinical experience, and one each for position and work pattern, as well as five psychological scores. This planning specification differs from the 10 predictor parameters in the final analysis.

Data were collected from October 27 to December 31, 2025, using SurveyMonkey. Recruitment began only after the institutional review board had approved the recruitment materials. Recruitment announcements were posted on online platforms used by nurses and on the groupware bulletin board of one tertiary general hospital with which none of the authors was affiliated. The announcement was posted after a formal request to the hospital was approved through its internal procedure. Participants were recruited through an open invitation and self‐selected into the study, yielding a convenience sample of 223 nurses. There was no defined sampling frame or eligible denominator from which participation rate could be calculated.

### 2.3. Instruments

A structured self‐report questionnaire was used to measure general and work‐related characteristics, self‐conscious emotions, mentalization, and colleague solidarity. The authors obtained permission to use all instruments included in this study before data collection.

#### 2.3.1. General and Work‐Related Characteristics

Self‐reported characteristics included age, sex, religion, education, total clinical experience, position, and work pattern.

#### 2.3.2. Self‐Conscious Emotions

The TOSCA‐3S [11] presents 11 negative everyday scenarios with four response statements per scenario (44 items), representing shame‐proneness, guilt‐proneness, externalization, and detachment. Responses range from 1 (*not likely*) to 5 (*likely*), and each 11‐item subscale has a possible total score of 11–55. Higher scores indicate stronger endorsement of the corresponding response tendency. Shame‐ and guilt‐proneness were used in the primary analysis. We used the Korean version of the TOSCA‐3S [20], which was produced through forward translation, independent bilingual back‐translation, reconciliation by the translators and authors, and review by an eight‐member content validity panel. In a study of 522 South Korean nurses using this version, Cronbach’s *α* ranged from 0.67 to 0.83 across the four subscales [13]; two‐week test–retest intraclass correlation coefficients were 0.85 for shame‐proneness and 0.87 for guilt‐proneness [20]. In a scenario involving a missed lunch appointment, an example of the shame response is “You would think, ‘I’m inconsiderate,’” while an example of the guilt response is “You would try to make it up to him as soon as possible.” In this study, Cronbach’s *α* was 0.75 for shame (*n* = 205) and 0.68 for guilt (*n* = 203, participants with complete guilt items); externalization and detachment had *α* values of 0.73 and 0.70 and were used only in sensitivity analyses.

#### 2.3.3. Mentalization

Mentalization was measured using the Korean version of the Mentalization Scale [21], adapted from Dimitrijević et al.’s original [18]. The instrument includes 25 items: 11 on other‐related mentalization, 6 on self‐related mentalization, and 8 on motivation to mentalize. Each item is rated on a 5‐point Likert scale ranging from 1 (*completely incorrect*) to 5 (*completely correct*), with total scores ranging from 25 to 125. Cronbach’s *α* was 0.84 for the total scale and ranged from 0.74 to 0.79 for the subscales in the original study [18]. In the Korean version, Cronbach’s *α* was 0.88 for the total scale and ranged from 0.74 to 0.84 for the subscales [21]. In this study, Cronbach’s *α* was 0.89 for the total scale, 0.86 for other‐related mentalization, 0.82 for self‐related mentalization, and 0.74 for motivation to mentalize. Items include statements such as “I can recognize other people’s feelings.”

#### 2.3.4. Colleague Solidarity

Colleague solidarity was measured using Kong and Shin’s Colleague Solidarity Scale [22]. This 31‐item instrument uses a 5‐point agreement scale (1 = strongly disagree to 5 = strongly agree), with total scores ranging from 31 to 155. Items 20–25 assess barriers to solidarity and are reverse‐scored before summing all items; higher totals indicate greater solidarity. One item reads, “There is a sense of camaraderie and loyalty among colleagues in our department.” Cronbach’s *α* was 0.94 for the original instrument and 0.96 in this study.

### 2.4. Data Analysis

Descriptive statistics summarized participant characteristics and scale totals. Pearson zero‐order correlations were calculated for the primary psychological variables and colleague solidarity. Partial correlations between guilt‐proneness and solidarity controlling for shame‐proneness, and between shame‐proneness and solidarity controlling for guilt‐proneness, were estimated from residual covariances. Mplus 9.1 robust maximum likelihood estimation provided robust standard errors, Wald *p* values, and 95% confidence intervals (CIs); partial‐correlation intervals used the delta method. Estimates and their uncertainty were combined across the 50 multiply imputed datasets described as follows.

Demographic and work‐related covariates were entered in hierarchical regression analysis first, followed by mentalization, guilt‐proneness, and shame‐proneness. Covariates were selected on conceptual grounds without bivariate significance screening. Clinical experience reflects accumulated clinical exposure; sex and religion represent social and value‐related context; and education, position, and work pattern reflect professional preparation, responsibilities, and opportunities for contact with colleagues. Clinical experience was retained and age excluded because of multicollinearity between the two. The final model contained seven covariate terms and three psychological predictors. *R* ^2^, adjusted *R* ^2^, and Δ*R* ^2^ were averaged across the 50 completed datasets. Pooled robust Wald tests assessed the overall slopes and each newly added psychological predictor.

Two participants had a missing response to the same guilt‐proneness item; no other value was missing. We generated 50 multiple imputations in Mplus with a Bayesian model that treated the missing item as ordered categorical and included the remaining guilt items, the other study variables, all covariates, and age as an auxiliary variable, assuming missingness at random; guilt totals were recalculated in each completed dataset, and a complete‐case analysis (*n* = 203) assessed sensitivity to imputation. Regression diagnostics were examined across the completed datasets: studentized residuals, leverage, Cook’s distances, variance inflation factors, residual plots, studentized Breusch–Pagan and Jarque–Bera statistics, and a pooled Wald test of quadratic terms. Sensitivity analyses added age, substituted age for clinical experience, recoded education (master’s degree or higher versus below) and clinical experience (< 5, 5–< 10, 10–< 15, and ≥ 15 years), added externalization and detachment, used conventional maximum likelihood, and excluded influential observations (Cook’s distance > 4/205 in any completed dataset). Common‐method variance was screened with a Harman single‐factor test of the 78 items measuring the four primary constructs, in the 203 complete cases and across the imputed datasets. Hospital identifiers were unavailable, precluding adjustment for hospital‐level clustering.

### 2.5. Ethical Considerations

This study was approved by the Institutional Review Board of Seoul National University (IRB No. 2508/001‐017; initial approval: July 31, 2025). An amendment to the recruitment materials was approved on October 27, 2025; the survey link was posted only after this approval had been received, and no responses were collected before then. This study was conducted in accordance with the principles of the Declaration of Helsinki. Nurses received a document explaining the study objectives, procedures, data collection methods, voluntary nature of participation, anonymity, and confidentiality. All participants gave online informed consent by clicking an agreement button before beginning the survey. Participants were informed that they could withdraw from the study at any time. Anonymity was maintained by not collecting personal identifiers, such as names or institutional affiliations.

## 3. Results

### 3.1. Participant Characteristics

Of the 223 respondents, 18 were excluded because they did not meet the inclusion criterion of working in a tertiary general hospital, leaving 205 for analysis. The 205 participants had a mean age of 35.40 years (standard deviation [SD] = 6.45) and mean clinical experience of 10.64 years (SD = 6.45). Most were female (93.7%) and staff nurses (89.8%; Table 1).

**TABLE 1:** Participant’s characteristics (*N* = 205).

Characteristics	Category	*n* (%)	*M* (SD)
Age (years)	< 30	34 (16.6)	35.40 (6.45)
	30–39	129 (62.9)	
	≥ 40	42 (20.5)	

Sex	Female	192 (93.7)	
	Male	13 (6.3)	

Religion	No	116 (56.6)	
	Yes	89 (43.4)	

Education	Three‐year college	2 (1.0)	
	Bachelor’s degree	154 (75.1)	
	Master’s or higher	49 (23.9)	

*Total clinical experience (years)*			10.64 (6.45)
Position	Staff nurse	184 (89.8)	
	Charge nurse	21 (10.2)	

Work pattern	Shift work	133 (64.9)	
	Fixed day work	72 (35.1)	

*Note: M*, mean. Percentages may not total 100 because of rounding. The standard deviations of age (6.449) and total clinical experience (6.448) coincide when rounded to two decimal places; both were verified against the raw data.

Abbreviation: SD, standard deviation.

### 3.2. Descriptive Statistics and Correlations Among Variables

Descriptive statistics are presented in Table 2; the solidarity item mean was 3.56 on a five‐point scale. Solidarity correlated negatively with shame‐proneness (*r* = −0.27) and positively with guilt‐proneness (*r* = 0.22) and mentalization (*r* = 0.41). Controlling for shame‐proneness, the guilt–solidarity partial correlation was 0.27 (95% CI: [0.16, 0.39], *p* < 0.001). Controlling for guilt‐proneness, the shame–solidarity partial correlation was −0.31 (95% CI: [‐0.46, −0.16], *p* < 0.001; Table 2).

**TABLE 2:** Descriptive statistics and correlations among variables (*N* = 205).

Variable	M (SD)	Min–max	Skewness	Kurtosis	1	2	3	4
1. Shame‐proneness	32.21 (7.14)	11–49	−0.20	−0.33	1			
2. Guilt‐proneness	46.17 (4.89)	29–55	−0.80	0.96	0.16 (0.006)	1		
3. Mentalization	94.08 (10.95)	60–125	0.26	0.30	−0.21 (0.006)	0.32 (< 0.001)	1	
4. Colleague solidarity	110.33 (19.04)	51–155	−0.30	0.39	−0.27 (0.001)	0.22 (0.001)	0.41 (< 0.001)	1

*Note:* Values are mean (SD) or correlation (p), combined across 50 imputations using Mplus robust maximum likelihood; descriptive means, SDs, skewness, and kurtosis are averages across completed datasets. Solidarity uses reverse‐scored Items 20–25. Min–Max, observed minimum and maximum; possible ranges are 11–55 for shame‐proneness and guilt‐proneness, 25–125 for mentalization, and 31–155 for colleague solidarity. Partial correlations with colleague solidarity (robust delta‐method inference, not adjusted for demographic covariates): guilt‐proneness controlling for shame‐proneness, *r* = 0.27 (95% CI: [0.16, 0.39], *p* < 0.001), and shame‐proneness controlling for guilt‐proneness, *r* = −0.31 (95% CI: [−0.46, −0.16], *p* < 0.001). M, mean. The *p* values for correlations are Wald tests of the standardized estimates with robust standard errors, pooled across imputations; conventional Pearson *t*‐tests (R 4.6.1) yielded *p* = 0.023 for the shame–guilt correlation and *p* ≤ 0.002 for the other correlations.

Abbreviations: CI, confidence interval; SD, standard deviation.

### 3.3. Adjusted Associations With Colleague Solidarity

The final model explained 26% of the variance in solidarity (adjusted *R* ^2^ = 0.22; Table 3). Mentalization (*B* = 0.50, *β* = 0.29, 95% CI: [0.11, 0.46], *p* = 0.001) and guilt‐proneness (*B* = 0.63, *β* = 0.16, 95% CI: [0.04, 0.29], *p* = 0.007) showed positive associations with solidarity, and shame‐proneness showed a negative association (*B* = −0.65, *β* = −0.25, 95% CI: [‐0.40, −0.09], *p* = 0.002); the CIs refer to standardized coefficients, and full estimates are given in Supporting Table S1. The added‐term Wald tests yielded *p* < 0.001 for mentalization, *p* = 0.103 for guilt before shame was entered, and *p* = 0.002 for shame. Covariate coefficients were imprecise; the estimate for male sex (*B* = 6.40, *p* = 0.050) rests on 13 participants and warrants cautious interpretation.

**TABLE 3:** Hierarchical regression of colleague solidarity (*N* = 205).

Variable	Model 1	Model 2	Model 3	Model 4		
	*β*	*β*	*β*	*β*	95% CI for *β*	VIF
Clinical experience	0.07	0.07	0.08	0.10	[−0.04, 0.23]	1.29
Sex: male	0.41	0.23	0.29	0.34	[0.00, 0.67]	1.06
Religion: yes	−0.13	−0.09	−0.08	−0.09	[−0.35, 0.17]	1.03
Education: 3‐year college	−0.78	−0.45	−0.59	−0.49	[−1.63, 0.64]	1.09
Education: master’s or higher	0.14	0.13	0.11	0.10	[−0.14, 0.35]	1.12
Position: charge nurse	0.39	0.29	0.25	0.30	[−0.04, 0.63]	1.17
Work pattern: fixed day	−0.12	−0.03	−0.02	−0.04	[−0.31, 0.23]	1.10
Mentalization	—	0.39^∗∗∗^	0.36^∗∗∗^	0.29^∗∗^	[0.11, 0.46]	1.27
Guilt‐proneness	—	—	0.10	0.16^∗∗^	[0.04, 0.29]	1.27
Shame‐proneness	—	—	—	−0.25^∗∗^	[−0.40, −0.09]	1.13
*R* ^2^	0.05	0.19	0.20	0.26		
Adjusted *R* ^2^	0.01	0.16	0.17	0.22		
Δ*R* ^2^	—	0.15	0.01	0.05		
Overall Wald *χ* ^2^ (df)	10.38 (7)	47.74 (8)	60.26 (9)	84.17 (10)		
Overall p	0.168	< 0.001	< 0.001	< 0.001		
Added‐term Wald *χ* ^2^ (1)	—	25.53	2.66	9.30		
Added‐term p	—	< 0.001	0.103	0.002		

*Note:* Model 1: demographic and work covariates; Model 2: add mentalization; Model 3: add guilt‐proneness; Model 4: add shame‐proneness. All models include an intercept and the same 205 participants. *β*, standardized coefficient (Mplus STDYX standardization for continuous predictors; STDY standardization for binary indicators); the 95% CIs refer to standardized coefficients in Model 4. Asterisks denote two‐tailed *p* values for the unstandardized coefficients, as reported in Supporting Table S1: ^∗^ *p* < 0.05, ^∗∗^ *p* < 0.01, and ^∗∗∗^ *p* < 0.001. Estimation used Mplus robust maximum likelihood with 50 item‐level multiple imputations. Reference categories: sex, female; religion, no; education, bachelor’s degree; position, staff nurse; and work pattern, shift work. Overall and added‐term Wald tests use pooled coefficients from 50 imputations. *R* ^2^, adjusted *R* ^2^, Δ*R* ^2^, and VIFs are averages across completed datasets. Clinical experience was retained instead of age because of multicollinearity.

Abbreviations: CI, confidence interval; VIF, variance inflation factor.

Residual diagnostics indicated departures from normality, without clear evidence of heteroscedasticity or jointly significant quadratic terms (*p* = 0.715). The directions and significance of the three psychological associations were consistent in complete‐case analyses, after excluding influential observations, and under alternative age adjustments. However, when externalization and detachment were included, the shame association was no longer statistically significant (*p* = 0.101), while mentalization and guilt remained positively associated with solidarity (Table 4). In the Harman screen, the first component explained 22.2% of item variance in complete cases and an average of 22.0% across the 50 imputed datasets.

**TABLE 4:** Sensitivity analyses for the psychological predictors.

Model	*N*	Mentalization *B* (*p*)	Shame *B* (*p*)	Guilt *B* (*p*)
Primary model: clinical experience	205	0.50 (0.001)	−0.65 (0.002)	0.63 (0.007)
Conventional maximum likelihood	205	0.50 (< 0.001)	−0.65 (< 0.001)	0.63 (0.017)
Add age	205	0.50 (0.001)	−0.65 (0.003)	0.64 (0.007)
Age instead of clinical experience	205	0.50 (0.001)	−0.66 (0.002)	0.62 (0.009)
Education: master’s or higher	205	0.51 (0.001)	−0.66 (0.002)	0.61 (0.010)
Categorical clinical experience	205	0.51 (0.001)	−0.65 (0.002)	0.65 (0.006)
Add externalization and detachment	205	0.48 (0.001)	−0.42 (0.101)	0.51 (0.041)
Exclude two missing‐item cases	203	0.50 (0.001)	−0.67 (0.002)	0.62 (0.009)
Exclude Cook’s distance flags	193	0.61 (< 0.001)	−0.64 (< 0.001)	0.65 (0.001)

*Note:* All models pool 50 imputations except the complete‐case model (*n* = 203) and use robust maximum likelihood except the conventional maximum likelihood model. The Cook’s distance model excludes the union of 12 flagged observations across imputations and omits the three‐year‐college indicator because both nurses in that category were excluded. Externalization mean = 25.30 (standard deviation = 6.94) and detachment mean = 29.65 (standard deviation = 6.26). B, unstandardized coefficient.

## 4. Discussion

This study examined whether shame‐proneness, guilt‐proneness, and mentalization were associated with colleague solidarity among nurses in South Korean tertiary general hospitals. After adjustment for demographic and work‐related characteristics, mentalization and guilt‐proneness were positively associated with solidarity, and shame‐proneness was negatively associated with solidarity in the primary model. Mentalization showed the largest association. The model accounted for about one‐quarter of the variance in solidarity, indicating that organizational and other unmeasured factors also matter. These cross‐sectional, between‐person associations identify psychological characteristics that warrant attention; they do not show that reducing shame‐proneness or increasing mentalization would directly improve solidarity.

The negative shame‐proneness association is compatible with the withdrawal and negative self‐evaluation represented by TOSCA‐3S responses [9, 10]. The association was moderate in size, but its interval estimate was wide. Solidarity could also shape emotional experiences, or unmeasured workplace conditions could be associated with both. Moreover, adding externalization and detachment attenuated the shame result. This sensitivity limits claims that shame‐proneness has a stable independent association across plausible models.

The comparison with earlier workplace research requires attention to the direction of the associations studied and to the level of measurement (state versus disposition). Burmeister et al. measured guilt and shame as feelings reported after hiding knowledge, in a hypothetical scenario and in a recalled workplace episode [15]. Their design therefore concerns emotional responses to a specific act, not whether a stable shame disposition predicts withholding. Miranda et al. examined shame after witnessing incivility and reported avoidance of both the target and instigator, together with withdrawal from work [16]. These studies support a link between shame and interpersonal disengagement.

The state–disposition distinction also matters for interpreting the TOSCA‐3S measure. Shame commonly involves a negative evaluation of the whole self, whereas guilt focuses on a specific behavior and its consequences [23]. The present result concerns shame‐proneness and does not imply that every episode of shame is harmful or should be eliminated. A meta‐analysis found that shame was more positively associated with a constructive approach when the failure or threatened social image was perceived as reparable [24]. Thus, its implications may depend on context and perceived opportunities for repair. Recognizing and addressing shame can also be difficult: shame may remain concealed or unacknowledged, and practitioners may avoid discussing it in clinical and therapeutic encounters [25]. Shame has long been described as a “sleeper” emotion that is easily overlooked [26], and it is often expressed indirectly, through anger or withdrawal. Thus, it must be inferred from discourse and behavior rather than from direct disclosure [27]. Professional experience alone should therefore not be assumed to ensure its recognition or effective management. The present coefficient should be used neither to label nurses as poor colleagues nor to locate responsibility for strained workplace relationships solely within the individual.

The positive zero‐order, shame‐adjusted, and final‐model associations for guilt‐proneness are compatible with the reparative orientation captured by TOSCA‐3S guilt responses [12]. The association was small and estimated with considerable uncertainty. Adjustment for shame does not directly measure a distinct shame‐free guilt trait. The results should not be extended to chronic or dysfunctional guilt, which the instrument did not assess.

After adjustment for the covariates and mentalization, guilt‐proneness was not significantly associated with solidarity before shame‐proneness was entered, but its coefficient increased and became statistically significant once shame‐proneness was added. This pattern is consistent with a statistical suppression effect: shame‐ and guilt‐proneness scores were positively correlated but related to solidarity in opposite directions, so controlling for shame removed variance that had partly obscured the positive guilt association [28]. The guilt result does not rest on this adjustment alone: guilt‐proneness was positively correlated with solidarity before any adjustment. Its shame‐adjusted coefficient also remained positive when externalization and detachment were included and in the influence‐screened analysis. The modest internal consistency of the guilt scale and the concentration of its scores near the ceiling would, to the extent that they reflect random error and restricted range, tend to attenuate rather than inflate the observed association, although shared self‐report variance could still inflate it. These measurement limitations also reduce precision, so the size of the guilt association should be interpreted cautiously.

The organizational literature provides possible contexts for future research. In a daily diary study of coworker pairs, Halbesleben and Wheeler examined reciprocal relationships between perceived support, trust, and helping [29]. Such work suggests examining whether opportunities for mutual support accompany the expression of reparative intentions. Future studies could combine dispositional guilt measures with reports of actual helping and repair, repeated assessments of support, and organizational measures, while keeping adaptive guilt distinct from chronic or dysfunctional guilt.

The positive mentalization association remained evident in the sensitivity model that included all four TOSCA‐3S response tendencies. The association was moderate in size and should be interpreted cautiously, as no clinically meaningful threshold has been established. Understanding others’ perspectives is a plausible link with solidarity [17, 19], but this study did not test that process. Further research should examine whether mentalization contributes to understanding nursing relationships beyond measured workplace conditions.

Domingo‐Osle and Domingo describe solidarity in terms of shared responsibility for care and attention to the needs of patients and the nursing team [30]. Understanding colleagues’ intentions and recognizing one’s own assumptions may help explain how mentalization relates to these responsibilities. Hayden et al. examined mentalization in relation to attachment and interpersonal distress in a clinical sample [31], providing another context for studying such relational processes. These conceptual and empirical links inform hypotheses about nursing relationships.

Clinical experience and age were strongly related. Sensitivity analyses adding age or substituting it for experience yielded consistent psychological associations. However, retaining clinical experience alone does not eliminate residual age‐related confounding. The small three‐year‐college group also limits education comparisons.

Several limitations constrain interpretation. First, the self‐selected online convenience sample may differ from nonparticipants in workplace experiences, emotional characteristics, or ability and willingness to complete an online survey. Nurses from the hospital whose groupware bulletin board carried the announcement may also be overrepresented. The sampling denominator and participation rate were unavailable. Participants were predominantly female and staff nurses in South Korean tertiary general hospitals; applicability to male nurses, nursing leaders, other hospital types, or other countries remains uncertain.

Second, all principal measures came from the same participants at one time. Common‐method effects, socially desirable responding, inaccurate self‐report, or conceptual overlap may have influenced the associations. Hypothetical TOSCA‐3S responses may differ from behavior in clinical situations. The relatively low internal consistency of guilt‐proneness warrants caution. The Korean TOSCA‐3S has published internal‐consistency evidence in nurses [13], but its factor structure and construct validity have not yet been published in peer‐reviewed form [20]. The shame–guilt correlation in this sample was lower than the moderate correlations usually reported for the TOSCA‐3S [10, 12] and the correlation observed in an earlier Korean nurse sample [13]; guilt scores were concentrated near the scale ceiling, which restricts range and attenuates correlations, so whether the low overlap reflects sample‐specific response patterns or measurement limitations cannot be determined. The imputation assumes missingness at random conditional on the model variables; the small number of missing responses does not establish that assumption. Multiple information sources, including colleague assessments, would strengthen future studies. The absence of a dominant first component in the Harman screen does not rule out common‐method bias, because this procedure has limited diagnostic value [32].

Third, the cross‐sectional design does not establish temporal ordering, mediation, or causal relationships. Reverse or reciprocal associations and residual confounding remain possible. Organizational conditions, leadership, staffing, emotional intelligence, and other unmeasured characteristics may be relevant; the unexplained variance cannot be assigned to any one of them. Hospital identifiers were not collected to preserve anonymity, so neither the distribution of participants across hospitals nor hospital‐level clustering could be evaluated. The sensitivity of the shame association to model specification also limits interpretation.

## 5. Conclusions

This study extends research on colleague solidarity, which has concentrated largely on organizational conditions, by showing that nurses’ mentalization and self‐conscious emotional tendencies are associated with the solidarity they experience with colleagues. Among 205 nurses in South Korean tertiary general hospitals, those with higher mentalizing capacity and a reparative guilt orientation reported stronger solidarity after adjustment for demographic and work‐related characteristics, and these associations were consistent across sensitivity analyses. Shame‐proneness was associated with weaker solidarity in the primary model, but this association was not statistically significant after externalization and detachment were added to the model and requires confirmation. Mentalization showed the largest and most consistent association. These results position mentalization and the shame–guilt distinction as individual‐level factors that complement organizational approaches to building collegial relationships in hospitals. They also suggest that how nurses interpret their own mistakes and their colleagues’ intentions is part of the fabric of solidarity, alongside working conditions. As the data are cross‐sectional and self‐reported, longitudinal and intervention studies are needed to determine whether strengthening mentalization and providing shame‐sensitive support can enhance solidarity, and to clarify how these characteristics interact with workload, leadership, and team climate. Such work would help nursing management move from describing solidarity to cultivating it.

## 6. Implications for Nursing Management

Nurses’ emotional and reflective capacities offer nursing managers a practical complement to organizational strategies for strengthening colleague solidarity. Solidarity is expressed in everyday conduct: how nurses offer assistance, acknowledge one another, and share responsibility for care. As mentalization showed the largest association, managers could build reflective practice into routines such as handovers, case conferences, error reviews, and preceptor–new nurse meetings, inviting nurses to consider a colleague’s intentions and their own reactions before responding to difficult interactions. Such practice requires both facilitators who model perspective‐taking and protected time in which reflection is expected rather than displaced by workload.

The opposite associations of shame‐ and guilt‐proneness in the primary model suggest that nurses’ characteristic responses to their own mistakes are relevant to solidarity. Brief psychoeducation could help nurses distinguish guilt about a specific action and its consequences from shame directed at the whole self, and case‐based reflection could then explore how a distressing moral experience can inform proportionate responsibility and feasible repair. Shame itself should not be treated as desirable, and humiliation should never be used to motivate staff; feedback after errors should address what happened, its consequences, and what can be repaired, while preserving the nurse’s dignity. As shame is readily concealed, managers should learn to recognize it, avoid shaming, and respond within emotionally safe relationships. Participation in reflective activities should never require public disclosure of distressing experiences. Linking this emotional distinction with adaptive coping, including problem‐solving, seeking support, and identifying realistic corrective actions, is a reasonable focus for education. Nurses experiencing persistent or overwhelming shame or guilt need access to confidential professional support beyond the manager’s role.

These individual‐level approaches should complement, not replace, attention to workload, staffing, leadership, and opportunities for mutual support, including conditions that support nurses’ autonomy, competence, and relatedness at work. The modest explained variance cautions against relying on individual training alone, and supportive relationships can coexist with difficult interactions, so management assessment should attend to everyday working relationships as well as overall solidarity scores. Staff discussions about access to help, communication, and structural constraints can identify local priorities. Whether such programs change mentalization or emotional responses, and whether any change is accompanied by greater solidarity, has not been tested; feasibility, acceptability, unintended distress, resource needs, and effects on solidarity should be evaluated before wider implementation.

## Author Contributions

Jiyeon Kim: conceptualization, methodology, investigation, data curation, and writing–original draft. Hyojung Choi: investigation, data curation, and writing–original draft. Sun Joo Jang: conceptualization, methodology, formal analysis, supervision, funding acquisition, writing–original draft, and writing–review and editing.

## Funding

This work was supported by a National Research Foundation of Korea grant funded by the Ministry of Science and ICT (Grant no. RS‐2025‐20563000).

## Disclosure

The funder had no role in the design of the study; in the collection, analysis, or interpretation of data; in the writing of the manuscript; or in the decision to publish the results. All authors approved the final version of the manuscript.

## Conflicts of Interest

The authors declare no conflicts of interest.

## Supporting Information

Additional supporting information can be found online in the Supporting Information section.

## Supporting information


**Supporting Information 1** Table S1. Hierarchical regression of colleague solidarity: full estimates (unstandardized coefficients, robust standard errors, *p* values, 95% CIs, and variance inflation factors).

**Supporting Information 2** STROBE_checklist_R3.

## Data Availability

The data that support the findings of this study are available from the corresponding author upon reasonable request.
