# Supplementary material for: Factors Associated With Colleague Solidarity Among Hospital Nurses: Shame‐Proneness, Guilt‐Proneness, and Mentalization
Source: J Nurs Manag. 2026 Nov 14;2026:4832980. doi: 10.1155/jonm/4832980 (PMC13768846; doi:10.1155/jonm/4832980)
Supplement: Supplementary file 1 [file JONM-2026-4832980-s001.docx]

**Table S1. Hierarchical Regression of Colleague Solidarity: Full Estimates (N = 205)**

| **Variable** | **Model 1** | | | **Model 2** | | | **Model 3** | | | **Model 4** | | | | |
| --- | --- | --- | --- | --- | --- | --- | --- | --- | --- | --- | --- | --- | --- | --- |
|  | **B (SE)** | **β** | **p** | **B (SE)** | **β** | **p** | **B (SE)** | **β** | **p** | **B (SE)** | **β** | **p** | **95% CI for B** | **VIF** |
| Clinical experience | 0.20 (0.22) | .07 | .361 | 0.20 (0.20) | .07 | .312 | 0.24 (0.20) | .08 | .239 | 0.29 (0.21) | .10 | .160 | [-0.11, 0.69] | 1.29 |
| Sex: male | 7.75 (4.93) | .41 | .116 | 4.28 (3.26) | .23 | .189 | 5.48 (3.24) | .29 | .091 | 6.40 (3.26) | .34 | .050 | [0.01, 12.78] | 1.06 |
| Religion: yes | -2.49 (2.70) | -.13 | .357 | -1.74 (2.54) | -.09 | .495 | -1.51 (2.55) | -.08 | .556 | -1.70 (2.50) | -.09 | .498 | [-6.60, 3.21] | 1.03 |
| Education: three-year college | -14.82 (14.99) | -.78 | .323 | -8.48 (12.65) | -.45 | .503 | -11.14 (13.02) | -.59 | .392 | -9.39 (11.04) | -.49 | .395 | [-31.02, 12.24] | 1.09 |
| Education: master’s or higher | 2.75 (2.80) | .14 | .326 | 2.54 (2.46) | .13 | .301 | 2.17 (2.52) | .11 | .390 | 1.99 (2.41) | .10 | .410 | [-2.73, 6.71] | 1.12 |
| Position: charge nurse | 7.32 (3.91) | .39 | .061 | 5.55 (3.11) | .29 | .074 | 4.70 (3.14) | .25 | .134 | 5.68 (3.30) | .30 | .085 | [-0.79, 12.15] | 1.17 |
| Work pattern: fixed day | -2.23 (2.97) | -.12 | .453 | -0.49 (2.72) | -.03 | .856 | -0.43 (2.70) | -.02 | .874 | -0.79 (2.61) | -.04 | .762 | [-5.90, 4.32] | 1.10 |
| Mentalization | — | — | — | 0.68 (0.14) | .39 | < .001 | 0.62 (0.14) | .36 | < .001 | 0.50 (0.15) | .29 | .001 | [0.20, 0.79] | 1.27 |
| Guilt-proneness | — | — | — | — | — | — | 0.40 (0.25) | .10 | .103 | 0.63 (0.24) | .16 | .007 | [0.17, 1.10] | 1.27 |
| Shame-proneness | — | — | — | — | — | — | — | — | — | -0.65 (0.21) | -.25 | .002 | [-1.07, -0.23] | 1.13 |
| R² | .05 |  |  | .19 |  |  | .20 |  |  | .26 |  |  |  |  |
| Adjusted R² | .01 |  |  | .16 |  |  | .17 |  |  | .22 |  |  |  |  |
| ΔR² | — |  |  | .15 |  |  | .01 |  |  | .05 |  |  |  |  |
| Overall Wald χ² (df) | 10.38 (7) |  |  | 47.74 (8) |  |  | 60.26 (9) |  |  | 84.17 (10) |  |  |  |  |
| Overall p | .168 |  |  | < .001 |  |  | < .001 |  |  | < .001 |  |  |  |  |
| Added-term Wald χ² (1) | — |  |  | 25.53 |  |  | 2.66 |  |  | 9.30 |  |  |  |  |
| Added-term p | — |  |  | <.001 |  |  | .103 |  |  | .002 |  |  |  |  |

Model 1: demographic and work covariates; Model 2: add mentalization; Model 3: add guilt-proneness; Model 4: add shame-proneness. All models include an intercept and the same 205 participants. B, unstandardized coefficient; SE, robust standard error; β, standardized coefficient (Mplus STDYX standardization for continuous predictors; STDY standardization for binary indicators). Two-tailed p-values refer to B; asterisks in Table 3 of the main text are based on these p-values. Estimation used Mplus robust maximum likelihood with 50 item-level multiple imputations. Reference categories: sex, female; religion, no; education, bachelor’s degree; position, staff nurse; work pattern, shift work.

Overall and added-term Wald tests use pooled coefficients from 50 imputations. R², adjusted R², ΔR², and VIFs are averages across completed datasets. Clinical experience was retained instead of age because of multicollinearity. The 95% CIs refer to unstandardized coefficients in Model 4.

CI, confidence interval; VIF, variance inflation factor.
